# Current Use of Asparaginase in Acute Lymphoblastic Leukemia/Lymphoblastic Lymphoma

**DOI:** 10.3389/fped.2022.902117

**Published:** 2022-06-30

**Authors:** Luke Maese, Rachel E. Rau

**Affiliations:** ^1^Huntsman Cancer Institute, University of Utah, Primary Children's Hospital, Salt Lake City, UT, United States; ^2^Department of Pediatrics, Baylor College of Medicine Texas Children's Hospital, Houston, TX, United States

**Keywords:** asparaginase, acute lymphoblastic leukemia, *Erwinia asparaginase*, therapeutic drug monitoring, hypersensitivity, pancreatitis

## Abstract

Pediatric Acute Lymphoblastic Leukemia (ALL) cure rates have improved exponentially over the past five decades with now over 90% of children achieving long-term survival. A direct contributor to this remarkable feat is the development and expanded understanding of combination chemotherapy. Asparaginase is the most recent addition to the ALL chemotherapy backbone and has now become a hallmark of therapy. It is generally accepted that the therapeutic effects of asparaginase is due to depletion of the essential amino acid asparagine, thus occupying a unique space within the therapeutic landscape of ALL. Pharmacokinetic and pharmacodynamic profiling have allowed a detailed and accessible insight into the biochemical effects of asparaginase resulting in regular clinical use of therapeutic drug monitoring (TDM). Asparaginase's derivation from bacteria, and in some cases conjugation with a polyethylene glycol (PEG) moiety, have contributed to a unique toxicity profile with hypersensitivity reactions being the most salient. Hypersensitivity, along with several other toxicities, has limited the use of asparaginase in some populations of ALL patients. Both TDM and toxicities have contributed to the variety of approaches to the incorporation of asparaginase into the treatment of ALL. Regardless of the approach to asparagine depletion, it has continually demonstrated to be among the most important components of ALL therapy. Despite regular use over the past 50 years, and its incorporation into the standard of care treatment for ALL, there remains much yet to be discovered and ample room for improvement within the utilization of asparaginase therapy.

## Introduction

Acute lymphoblastic leukemia (ALL) remains the most frequently diagnosed malignancy of childhood, accounting for 21% of all diagnoses (1). Since its initial description in the mid-nineteenth century the understanding of ALL biology and treatment has evolved. For the first 100 years after its discovery, ALL remained an almost universally fatal disease. It was not until the mid-twentieth century, with the advent of chemotherapeutics, that remission and longer-term survivals were documented. Asparaginase, first shown to have anti-tumor properties in the 1960's (2), is the most recent chemotherapeutic to become universally incorporated into the multi-agent backbone in the treatment of ALL. L-Asparaginase is an enzyme that hydrolyzes asparagine into aspartic acid and ammonia depleting the circulating pool of serum asparagine (3). Prolonged deprivation of asparagine leads to reduced protein synthesis and initiation of apoptosis. Additionally, normal cells may endogenously produce asparagine which, in turn, leads to selective killing of malignant cells by asparaginase as they lack asparagine synthetase.

Asparaginase is produced by many different organisms and for the incorporation into therapeutics it is most commonly derived from *Escherichia coli* (*E. coli*) and/or *Erwinia chrysanthemi*. In the late 1960's *E. coli* derived asparaginase was first incorporated into the treatment of malignancies, primarily lymphoma and leukemia, demonstrating dose dependent remissions (4). However, its greatest success has been as a component of a multiagent chemotherapeutic backbone, with inductions remission rates of 93% noted in original investigations (5). Over the past 50 years the importance of asparaginases in the treatment of ALL has been continually demonstrated. Whether it be directly comparing patients who received or did not receive asparaginase, or examining effectiveness of asparaginase intensification, outcomes remain superior in those given maximal asparaginase therapy (6–8).

The importance of asparaginase therapy in the treatment of ALL has spurned great developments in optimization of therapeutic drug monitoring (TDM). Asparaginase pharmacokinetics (PK) has become an important part of how the drug is incorporated into protocols. While asparagine is difficult to reliably measure due to rapid *ex vivo* breakdown, serum asparaginase activity (SAA) is more easily measured, and has correlated well with asparagine depletion and clinical effectiveness (9). TDM has allowed for optimization of dosing regimens for all asparaginase formulations. Optimizing asparaginase depletion also includes accounting for the toxicities which may limit use and lead to significant side effects. There are several strategies utilized for the incorporation of asparaginase therapy many of which will be reviewed here. Despite over a half century of asparaginase utilization in ALL therapy, there remains much to be learned and many avenues for improvement.

## Currently Available Products

Since the discovery of asparaginase over 60 years ago there have been a variety of drugs developed and approved by regulatory bodies (Table 1 details currently available asparaginase formulations). As with most drug development processes each generation of products has sought to improve upon previous versions. These improvements have centered around prolonging duration of effectiveness, augmenting safety profile, improving ease of administration, and modernizing manufacturing processes. *E. coli* and *Erwinia chrysanthemi* are the primary bacteria utilized for production of pharmaceutical asparaginase and serve to draw major distinctions between products. But most importantly, as the asparaginase landscape as evolved, pegylated molecules have become the first-line agents of choice for cooperative groups and treating clinicians throughout the world.

**Table 1:** Asparaginase formulations.

**Drug**	**Initial FDA approval**	**Half-life (days)**	**Recommended dosing and interval**	**Recommended interval**	**Seminal US trials**
Pegaspargase	1994	IM: 5.73	2,500 IU/m^2^ for patients	No more frequently	DFCI 91-01
(*Oncaspar®*)		IV: 5.3	≤21 years	than every 14 days	CCG-1962
			2,000 IU/m^2^ for patients		
			>21yrs		
Calaspargase pegol-	2018	IV: 13.4	2,500 IU/m^2^ (only	No more frequently	COG AALL07P4
mknl		IM: NA	approved or patients ≤ 21	than every 21 days	(NCT00671034)
(*Asparlas®*)			years)		DFCI 11-001
					(NCT01574274)
Asparaginase Erwinia	2011	IM: 0.65	25,000 IU/m^2^	3 times/week	COG AALL07P2
chrysanthemi		IV: 0.31		(M/W/F) for 6 doses	(NCT00537030)
(*Erwinaze®)*					DFCI 00-01
					(NCT00165178)
					DFCI IV trial
					(NCT01643408)
Asparaginase erwinia	2021	IM: 0.76	25 mg/m^2^	Every 48 hours (every	COG AALL1931
chrysanthemi		IV: TBD		72-hour dosing under	(NCT04145531)
(recombinant)-rywn				investigation)	
(Rylaze®*)*					

*IM, intramuscular; IV, intravenous; IU, International Units; M/W/F, Monday, Wednesday, Friday*.

Pegylation, the covalent and non-covalent attachment of Polyethylene glycol (PEG) moieties to molecules was first explored in the 1970's (10). This posttranslational modification process was a fortuitous development in asparaginase therapeutics as it improved upon two major deficiencies of the first generation of drugs; duration of action and immunogenicity (11). Prior to the development of pegylation, asparaginase products were only available in their native forms. Native *E. coli* derived asparaginase, commonly known as Elspar (Asparaginase medac, European preparation) and no longer available in North America, was the first asparaginase molecule developed for widespread clinical use. With a half-life of 1.24 days, in order to obtain sustained depletion of asparagine for an adequate period of time, multiple doses were required (12). Additionally, between 30 and 81% of patients experienced hypersensitivity reactions, significantly limiting the use of native *E. coli* derived asparaginases (7, 9, 13–15). These characteristics necessitated further development of asparaginase molecules which was first realized upon the discovery of pegylation in the form of pegaspargase.

The initial reports investigating the pharmacodynamics (PD) and PK of pegaspargase in human subjects occurred in the 1980's (16). A seminal report found dosing between 2,000 and 2,500 IU/m^2^ demonstrated a half-life of 357 +/- 243 h with a single dose capable of maintaining complete asparagine depletion for 2 weeks (16). Many small, early phase studies of pegaspargase followed in the setting of relapsed disease and/or hypersensitivity to native *E. coli* asparaginase, the majority of which demonstrated encouraging results (17–19) and contributed to the initial FDA approval in 1994 for the aforementioned indications (20). These were then followed by larger phase II and III trials, several of which served to establish pegaspargase as a mainstay of therapy in ALL. DFCI 91-10 compared the use of E. coli asparaginase to pegaspargase during a 30-week intensification and, while no difference in event free survival (EFS) was noted, patients tolerating at least 26 weeks of asparaginase had superior 5 year EFS (90 vs. 73%, *p* < 0.01) (21). The Children's Cancer group (CCG) conducted a similar phase II trial comparing native *E. coli* derived asparaginase to pegaspargase with the main goals of assessing differences in safety and antibody production (22). Results were notable for decreased incidence of antibodies and absence of antibodies associated with rapid clearance in patients receiving pegaspargase, in the setting of a similar safety profile to native *E. coli* asparaginase. These two trials contributed to an expanded approval for pegaspargase in 2006 for the first-line treatment of patients with ALL (20). More recently several large systemic reviews have reinforced these early findings and demonstrated superiority of pegaspargase in comparison to native *E. coli* asparaginase (23–25). Cost effectiveness of pegaspargase in comparison to native *E. coli* asparaginase has also been demonstrated (26).

Calaspargase pegol-mknl (Cal-PEG), a more recently introduced long-acting asparaginase now being incorporated into the therapy of ALL, is similar to pegaspargase but with some important differences. Derived from *E. coli* it contains a more stable succinimidyl carbonate (SC) linker region to the polyethylene glycol molecule, as opposed to the succinimidyl succinate (SS) linker region of pegaspargase. This linker contributes to several defining characteristics including a longer half-life and an extended shelf-life. Two landmark clinical trials contributed to FDA approval of Cal-PEG in December of 2018 (27). COG AALL07P4 was designed to determine the PK and PD comparability of pegaspargase to Cal-PEG (28). Newly diagnosed high-risk B-ALL patients were randomly assigned to receive Cal-PEG at two separate doses 2,100 (*n* = 69) or 2,500 IU/m^2^ (*n* = 42) or PEG at 2,500 IU/m^2^ (*n* = 54), to be incorporated into prescribed multi-agent chemotherapeutic backbone. In this direct comparison the Cal-PEG mean half-life of plasma asparaginase activity was 2.5x longer than pegaspargase. Concordantly, plasma asparagine was undetectable for 18 days in patients who received Cal-PEG vs. only 11 days in the pegaspargase cohort. Despite this longer period of depletion, the study demonstrated a comparable toxicity profile with notable increased incidence in hyperglycemia and hyperbilirubinemia during induction and delayed intensification phases respectively. While the response rates between 2,500 IU/m^2^ of pegaspargase and Cal-PEG were equivalent, the 2,100 IU/m^2^ Cal-PEG dosing demonstrated a trend toward lower rates of response which crossed predefined monitoring boundaries resulting in closure of this arm.

DFCI 11-001 also compared pegaspargase and Cal-PEG in the context of their multiagent chemotherapeutic backbone, which included a single induction dose along with a 30-week post-induction period of single agent asparaginase therapy (29). During this prolonged period of asparaginase therapy, patients were randomly assigned to receive either pegaspargase every 2 weeks for a total of 15 doses or Cal-PEG every 3 weeks for a total of 10 doses. Induction PK results demonstrated equivalent asparaginase activity at 18 days post-dose, however at 25 days Cal-PEG demonstrated superior activity (88 vs. 17%; *p* < 0.001). Post-induction asparaginase activity was equivalent, demonstrating less frequent dosing of Cal-PEG during prolonged period of asparaginase was feasible. Similar to AALL07P4, there were no significant differences in toxicity or disease response rates between Cal-PEG and pegaspargase.

While Cal-PEG does have sustained activity superior to pegaspargase, the optimal incorporation of it into treatment regimens remains ill-defined. While these two previously mentioned landmark studies showed no difference in outcomes, they were not powered to do so. In theory extended depletion of asparagine should be beneficial, but within a multiagent chemotherapeutic backbone this is difficult to quantify. Currently, looking at cycles of therapy where multiple doses of asparaginase are prescribed in close proximity, i.e., DFCI regimens, where in you could decrease the total number of doses thereby minimizing exposure and caregiver burden is an optimal place for Cal-PEG (30). Additionally, Cal-PEG is currently FDA approved for individuals <22 years of age and not approved for use outside of the United States (27).

Although, pegylated asparaginase products are preferred there are non-pegylated agents that remain available and are essential in the treatment of ALL. Asparaginase *Erwinia chrysanthemi* (ERW), is a non-*E. coli* derived asparaginase developed in the 1970's and used primarily in the setting of hypersensitivity to an *E. coli* derived product (31, 32). As it is derived from a different bacterial source, the products are immunologically distinct with no cross reactivity (33). ERW has similar pharmacokinetic properties to native *E. coli* asparaginase with a slightly shorter half-live of 0.65 days [when administered intramuscularly (IM)] (34). DFCI 00-01 included a cohort of newly diagnosed ALL patients who experienced hypersensitivity to *E. coli* asparaginase and were switched to IM ERW for all remaining courses of asparaginase therapy (35). They found ERW to be well-tolerated and most patients achieved effective asparaginase activity. Most importantly, there was no difference in event-free survival between the two cohorts of patients. COG AALL07P2 also evaluated the safety and efficacy of ERW in the setting of *E. coli* derived asparaginase hypersensitivity demonstrating similar findings of acceptable safety profile and activity levels (36). It was concluded that 6 doses of IM ERW were an acceptable substitute for one dose of pegaspargase. ERW was FDA approved in 2011 as an alternative for patients with ALL in the setting of *E. coli* derived hypersensitivity (31). However, beginning in 2016 manufacturing difficulties led to repeated instances of global supply shortages driving the need for additional non-*E. coli* derived products^1^.

The most recent asparaginase to receive FDA approval, asparaginase erwinia chrysanthemi (recombinant)-rywn (JZP-458 or Rylaze), is a recombinant *Erwinia* asparaginase produce in a novel *Pseudomonas* fluorescens expression platform. Rylaze has the same amino acid sequence as EWR and thus no immunologic cross-reactivity to *E. coli*-derived asparaginase (37). The recombinant manufacturing process will curtail any future production and supply issues that arose with ERW (37). COG AALL1931 (NCT04145531) is an ongoing phase 2/3 study of Rylaze in patients with ALL/LBL who developed hypersensitivity or silent inactivation to a long-acting *E. coli*–derived asparaginase. Preliminary results of this study demonstrated adequate asparaginase activity and a safety profile consistent with other asparaginase products. This led to an accelerated FDA approval of 25 mg/m2 administered IM at every 48-h intervals in June of 2021^2^. A primary objective of the trial included adequate asparaginase activity at a 72-h trough and additional dosing regimens were explored. A population PK model predicted adequate 72-h asparaginase levels with a dose of 25 mg/m^2^ on Monday and Wednesday and 50 mg/m^2^ on Friday, thus this was explored in a cohort of patients on AALL1931 (38).

While less important in pegylated asparaginase products as they display time-dependent elimination, route of administration plays a significant role in the pharmacokinetics of non-pegylated products (39). Erwinia asparaginase exhibits linear elimination and includes a rate-limiting step in the absorption phase (40, 41). When given IV, this step is bypassed resulting in faster elimination of drug, and hence more abrupt asparaginase activity (42). As a result, the half-lives of Erwinia have been calculated at ~16 and 6 h for the IM and IV route, respectively (13, 34, 36, 40).

## Toxicities

Asparaginase, like most cytotoxic chemotherapeutics, induce off-target effects leading to adverse side effects. Many of these toxicities overlap with other medications utilized in the treatment of ALL, however some are unique to asparaginases and warrant detailed discussion (Table 2).

**Table 2:** Prominent asparaginases toxicities.

**Toxicity**	**Rates**	**Risk factors**	**Mitigation technique(s)**	**Rechallenge**
Hypersensitivity	10-15%	• Prolonged gaps between doses • *CNOT3, NFATC2*, HLA genetic variants	• Premedication • Switching to alternative product • Desensitization	No; may consider in the setting of ≤ grade II hypersensitivity if suspect infusion reaction
Pancreatitis	5-10%	• Older age • *CPA2, ULK2, RGS6* genetic variants	• NA	Not recommended for severe grade 3 or any grade 4; consider for grade 1/2 or mild grade 3
Thrombosis	5%	• Older age • T-ALL • Increased exposure	• Thromboprophylaxis under investigation (NCT02369653 and NTR4707)	Yes; in the setting of appropriately treated and/or resolved thrombosis
Hepatotoxicity	Variable; Increased transaminases up to 60% Hyperbilirubinemia 5-10%	• Obesity • Hispanic ethnicity • *SOD2* genetic variant	• Hold in the setting of significant toxicity • Capped dosing for obese individuals • Levocarnitine and Vitamin B12 under investigation (NCT03564678)	Yes

Hypersensitivity reactions are perhaps the most important and most thoroughly studied side effects of asparaginase treatment, both due to their severity and implications for further treatment. As these drugs derived from bacteria are foreign proteins, they carry strong immunogenic potential. Additionally, the PEG moiety in selected asparaginase products also precludes the potential for an immunogenic response. There have been several reports indicating that antibodies to PEG and/or the linker region are more prominent than those to the asparaginase protein itself (43–45). Asparaginase hypersensitivity is antibody mediated but the detailed pathogenesis remains unclear. There is an association with formation of antigen-specific IgE and IgG antibodies, and while the detection of anti-asparaginase antibodies has been successful in some studies, it has not been universally predictive of hypersensitivity (9, 43, 46–48). Appropriate grading and classification of hypersensitivity is essential. As pegaspargase is now predominantly given *via* intravenous infusion, infusion-related reactions occur not uncommonly. Infusion reactions are not truly antibody-mediated, often mimic true-hypersensitivity reactions, and distinguishing between the two is often difficult (49). Timing of reaction (after the second or third exposure most common in true hypersensitivity), symptoms (angioedema, pathognomonic for true hypersensitivity), and therapeutic drug monitoring are all items that may give clues as to the underlying pathophysiology. ERW, while often substituted in patients with hypersensitivity to pegylated asparaginase, also carries the potential for immunogenicity. Clinical manifestations of reaction are more often less severe, consisting of local skin reactions (36, 50, 51).

Factors influencing hypersensitivity include formulation of asparaginase, timing of administration, utilization of mitigation techniques (i.e., pre-medication), and genetics. The increased incidence of hypersensitivity, as high as 75% in one report, seen with native *E. coli* asparaginase administration is not observed with pegylated asparaginase where rates are reported between 10 and 15% (14, 29, 43, 52, 53). Timing of administration with continuous periods of exposure with less gaps in treatment are associated with lower rates of hypersensitivity (52, 54). Various reports of mitigation techniques, including pretreatment with corticosteroids and antihistamines exist, many with mixed results (55–58). However, with limited downside premedication has become common practice in many institutions. It is important to note that while premedication may mitigate symptoms of hypersensitivity, the ability to decrease antibody mediated hypersensitivity reactions is as of yet undetermined, and the utility of this approach is likely in prevention of non-antibody mediated infusion reactions. Several genome wide association studies have demonstrated a variety of germline genetic variants including in *CNOT3, NFATC2*, and in the human leukocyte antigen (HLA) region associated with increased hypersensitivity rates, however these discoveries have yet to impact clinical practice (59, 60).

In the setting of a true antibody-mediated hypersensitivity reaction the first option for modification of treatment is switching to an alternate asparaginase formulation derived from a different bacterial protein (9, 61, 62). Switching to an alternative formulation has demonstrated equivalent outcomes when compared to those who complete full prescribed asparaginase courses with first line therapy (8). The aforementioned shortages in ERW prompted institutions in the last several years to attempt desensitization protocols to PEG, which have demonstrated mixed results (63–65). It is important to note that there is no data available on the potential impact of desensitization on survival outcomes. Additionally, a report from the DFCI demonstrated that in patients with grade 2 or less hypersensitivity to pegaspargase, approximately half may be rechallenged successfully with the addition of pre-medication (acetaminophen, diphenhydramine, and hydrocortisone) and a slower infusion rate (66).

Pancreatitis is an additional asparaginase toxicity that often carries drastic consequences for patients with an estimated associated mortality of 2% (67). The mechanism has been linked to asparaginase inhibition of protein synthesis and its effect on calcium and adenosine triphosphate (ATP) control of cellular pathology (68, 69). Its incidence, reported between 2 and 18%, is associated with cumulative exposure to asparaginase and is seen in greater frequency at older ages (67, 70, 71). However, there has been limited association of increased risk of pancreatitis with any of the FDA approved asparaginase formulations. Genotype-phenotype correlations have also been reported but have yet to impact clinical practice (71, 72). As pancreatitis may be diagnosed in several ways including through (1) clinical signs/symptoms (abdominal pain, emesis, nausea, back pain, fever), (2) laboratory based studies (amylase and/or lipase >3 times upper limit of normal), and (3) radiological imaging (ultrasound, CT, or MRI) an internationally agreed upon definition has been established stating two of these three characteristics must be present in order to diagnose asparaginase-associated pancreatitis (62). Historically due to the severity of initial pancreatitis, and potential for reoccurrence with additional exposure, rechallenging with further asparaginase therapy has been avoided. However, with the continued emphasis on asparaginase therapy, and the demonstration of its importance within treatment of ALL, recent investigations into rechallenging have proved successful in ~50% of patients (67, 70). While there has yet to be well-defined criteria for rechallenging, many groups consider it in the setting of all grade 2 pancreatitis and grade 3 pancreatitis without prolonged illness or severe complications.

Thrombosis or hemorrhage, either mild or severe, may be encountered in the setting of asparaginase therapy. While confounding factors such as concomitant active leukemia, glucocorticoid use, and indwelling central catheters exist, the known disruption of protein synthesis as a result of asparaginase effect on the proteins involved in the coagulation cascade and fibrinolysis is an established mechanism (73–75). As thrombosis is more common than clinically significant hemorrhage, there is much more known about the former. With modern therapeutic regimens the incidence of asparaginase-associated thrombosis in pediatric ALL ranges from 2 to 8%, with the most common location in the extremities, but with a significant proportion also occurring in the cerebral sinuses (75–77). Clinical characteristics associated with thrombosis include older age, T-ALL, and increased length of exposure to asparaginase (76, 77). Asparaginase therapy should be withheld during clinically significant thrombosis or hemorrhage and appropriate treatment should be initiated. Treatment may be resumed depending on severity, however cerebral sinus thrombosis carries increased risk for morbidity and mortality and may preclude additional asparaginase therapy. While there are ongoing studies investigating the role of thromboprophylaxis in pediatric ALL, it is not currently recommended (78–80).

Hepatotoxicity is an additional notable side effect of asparaginase therapy. The disruption of protein synthesis is likely causative, although oxidative stress has also been implicated (81). It is manifested in the form of non-cholestatic (elevated transaminases) and cholestatic (elevated bilirubin) abnormalities. Similar to other toxicities, overlap of concomitant chemotherapeutics that also result in hepatotoxicity may lead to difficultly ascertaining a primary cause. Significant elevation of transaminases (≥ grade 3) is quite common during multiagent chemotherapy, and is often expected, however rarely results in clinical morbidity. Notable cholestasis (≥ grade 3) has been seen in up to 9% of patients receiving asparaginase, but in the pediatric population this is not often clinically significant (82). There have been correlations with increased risk for hepatotoxicity including, obesity, genotype (*SOD2* rs4880 CC), and Hispanic ethnicity (82–84). Regardless, there are currently no guidelines on modification of asparaginase therapy in the pediatric population for hepatotoxicity and in the majority of cases therapy is not altered. Administration of Levocarnitine as a prevention for hepatoxicity has been piloted with encouraging results, however more robust prospective data is required to determine its potential utility (85).

While hypersensitivity, pancreatitis, coagulopathy, and hepatotoxicity are of the utmost importance, asparaginase treatment has other notable toxicities. Hyperammonemia, an often-encountered result of the mechanism of asparaginase as it drives the breakdown of asparagine to aspartic acid and ammonia, may mimic hypersensitivity and infusion reactions resulting in nausea, and emesis (86). However, the severity rarely rises to a grade 3/4 level and has not been definitively associated with clinically significant neurotoxicity (87). Two additional toxicities of asparaginase, hypertriglyceridemia and osteonecrosis, are also implicated in the setting of glucocorticoid use, another mainstay of ALL therapy. Grade 3/4 hypertriglyceridemia is encountered in up to 47% of patients and is predominantly seen in the setting of pegylated asparaginase (87). Despite this, it is rare to encounter secondary sequelae, i.e., pancreatitis, as a result of hypertriglyceridemia (87, 88). As osteonecrosis is frequently encountered at the end or after completion of therapy, it is difficult to draw definitive conclusions. Increased use of asparaginase has been postulated as potentiating the osteonecrosis effect of glucocorticoids (89). Additionally, despite efforts to mitigate osteonecrosis incidence with discontinuous use of glucocorticoids, there was no benefit realized which may have been attributed to an increased use in asparaginase therapy (90).

A particular challenge within administration of asparaginase lies within the adolescent and young adult (AYA) population. AYAs are at risk for many increased toxicities partly due to increased body surface area, and hence larger doses of asparaginase (91). To curtail this many treating oncologists have employed dose-capping of pegaspargase to 3,750 IU or 1 vial (92). This is an active area of investigation through which TDM and individualized dosing investigations are well-positioned to provide further insight.

### Current Use of Asparaginase in ALL/LBL

While asparaginase is a critical component of ALL/LBL therapy across all major pediatric and AYA cancer consortia, the dosing and administration schedule differs between groups. For example, some groups rely on intermittent doses of asparaginase throughout pre-maintenance therapy, whereas other groups aim for prolonged continuous asparagine depletion. Examples of these strategies can be found by examining the asparaginase dosing regimens of the three major pediatric cancer consortia in North America, the Children's Oncology Group (COG), Dana Farber Cancer Institute (DFCI) and St Jude Children's Research Hospital (SJCRH). These groups each employ different asparaginase strategies and continue to investigate refinement of asparaginase use in the context of their ALL/LBL protocols.

The COG largely relies on intermittent dosing of asparaginase interspersed throughout most blocks of premaintenance therapy, with the total number of doses varying based on the relative risk of relapse. Current COG ALL protocols for NCI standard risk (SR) B-ALL patients without higher risk features include only one dose of pegaspargase in induction and one in the post-induction phase of therapy (93). This minimalistic approach to asparaginase therapy was tested in the randomized COG study, AALL0331, in which patients with low-risk features were randomized to receive either four doses of PEG 2,500 IU/m^2^ IM given at 3-week intervals during consolidation and interim maintenance therapy or only one PEG 2,500 IU/m^2^ IM dose during delayed intensification (DI). PEG intensification did not prove superior to standard therapy with 5-year continuous complete remission rates for intensified PEG vs. standard therapy of 96.0 +/- 0.8% vs. 94.4 +/- 0.4%, and 5-year overall survival of 98.3 +/- 0.6% vs. 99.3+/-0.4% (94), suggesting in the context of the multiagent chemotherapy backbone, minimal asparaginase is necessary for cure for these low-risk patients. On the other hand, NCI high risk (HR) B-ALL patients and NCI SR B-ALL with higher risk features such as end of induction MRD positivity or unfavorable genetic features, receive up to eight doses of PEG across various treatment blocks. This intensified post-induction chemotherapy backbone is based on protocols pioneered by the Berlin-Frankfurt-Munster (BFM) consortium (95) but have been augmented including by adding doses of asparaginases during multiple phases of therapy. This augmented approach was first tested in randomized fashion by the Children's Cancer Group, finding that ALL patients with a slow early response to induction therapy (>25% blasts in bone marrow at day 7 of induction) had superior event free survival when treated with augmented post-induction chemotherapy with added asparaginase doses compared to those treated with the standard, non-augmented chemotherapy backbone (96). While the optimal number of PEG doses for the treatment of higher risk patients has not been definitively established, recent COG data demonstrated inferior outcomes for NCI HR patients when asparaginase courses are omitted, suggesting optimal outcomes require receipt of all planned doses on this intermittent dosing schedule (8).

In contrast to the intermitting dosing strategy, some consortia employ a chemotherapy backbone that includes continuous asparagine depletion for much of post-induction, pre-maintenance therapy. This strategy is largely based on work from the DFCI which currently incorporates 30 contiguous weeks of asparaginase depletion, based on results from the DFCI 91-01 study as described above (21). In their subsequent ALL trial, DFCI 05-001, patients were randomized to receive these 30-weeks of asparaginase therapy as either 30 weekly doses of native E. coli asparaginase 25,000 IU/m^2^ IM or 15 doses of PEG 2,500 IU/m^2^ IV given every other week. While the safety and efficacy of both asparaginase preparations was similar, there was significantly less anxiety associated with IV PEG administration, supporting its use over IM native *E. coli* asparaginase in the front-line setting (97).

The St Jude Children's Research Hospital (SJCRH) Consortium uses a risk-adapted asparaginase approach, in which patients with low-risk features receive intermittent post-induction doses and higher risk patients receiving continuous dosing. On SJCRH Total Therapy Study 16, for patients who were categorized as low risk based on cytogenetics and early response to therapy, 4 post-induction doses were included in their post-induction regimen. Patients categorized as standard or high risk on the other hand, received 15 doses of PEG given every other week. Of note, as Total 16 was the first SJCRH study to eliminate cranial irradiation for all patients, enhanced IT and systemic CNS directed therapy was studied, including the randomized study of the standard PEG dose of 2,500 IU/m^2^ vs. a higher dose of 3,500 IU/m^2^. The results of Total 16 showed that while omission of cranial irradiation was safe and feasible with appropriate intensification of IT therapy, the higher dose of PEG did not significantly improve outcomes (98). This result suggests that while the higher dose likely provides higher peak concentrations, 2,500 IU/m^2^ is adequate to maintain continuous serum and CSF asparagine depletion.

While outcomes on COG, DFCI and SJCRH studies are overall excellent and largely comparable, other factors must be considered. A recent analysis compared outcomes and health care utilization costs between Canadian centers using COG protocols (intermittent dosing) vs. those using DFCI protocols (continuous dosing). In adjusted analyses, the cost intensity of care was 70% higher among institutions using DFCI protocols, largely due to increased outpatient visits and chemotherapy costs due to more doses of PEG (99). To mitigate this cost difference, it is possible use of the longer acting CAL-PEG preparation may allow for fewer asparaginase doses to maintain asparagine depletion for the requisite time, translating into fewer outpatient clinic visits which could lessen these costs in the future. Additionally, as discussed below, use of therapeutic drug monitoring (TDM) to individualize dosing could result in lowered doses of pegylated asparaginases for many patients, resulting in lower chemotherapy costs.

Notably, retrospective comparisons of different studies run by different consortia using either intermittent or continuous asparaginase dosing in combination with different chemotherapy backbones is an inadequate method to determine if there are safety or efficacy difference between the two approaches. To overcome this, the recent Nordic Society of Pediatric Hematology and Oncology study, NOPHO ALL2008 study compared in a randomized fashion these dosing strategies for non-high-risk pediatric ALL patients. The NOPHO consortium typically uses a continuous dosing schedule. In ALL2008, after 10 weeks of continuous asparaginase therapy (five doses of PEG 1,000IU/m^2^ IV given every 2 weeks), patients were randomized to receive either three additional doses given at 6-week intervals (experimental arm, *N* = 309) or five more doses given every 2 weeks (standard arm, *N* = 316). The 5-year DFS was not significantly different between arms, 92.2% (95% CI 88.6–95.8) and 90.9% (95% CI 87–94.6) for the experimental intermittent dosing and standard continues dosing arms, respectively. However, the risk for target toxicities including hypersensitivity, osteonecrosis, pancreatitis and thromboembolism was significantly higher in the standard, continuous dosing arm. This article supports that for the population studied, intermittent dosing is efficacious and may reduce toxicity risk (100).

### Therapeutic Drug Monitoring

As the main therapeutic effect of asparaginases is a result of asparagine depletion, measurement of plasma asparagine would be the most direct way to determine if a patient achieved an adequate response to asparaginase therapy. However, while it is possible to measure plasma asparagine, it is cumbersome and challenging as a result of ongoing *ex vivo* hydrolysis in the presence of asparaginase in the serum. For results to be valid, extensive measures including placing the serum sample on ice immediately after collection and an addition of an asparaginase inhibitor to the collection tube, should be taken (101). Therefore, it is generally not feasible to accurately measure plasma asparagine outside the context of a clinical trial. To overcome these limitations, surrogate assays which quantify the serum asparaginase activity (SAA) have been developed. A number of studies have demonstrated a strong inverse correlation between SAA levels and serum asparagine leading to their acceptance as valid therapeutic drug monitoring assays (12, 102–105). SAA levels are now commercially available through two CLIA certified labs in the US.

While most experts would agree that to be considered a therapeutic SAA level, it must correlate with complete plasma (and perhaps CSF) asparagine depletion. However, at what level that is consistently achieved remains a topic of debate. In early work including rhesus monkeys and human samples, Riccardi et al. established that SAA levels of ≥0.1 IU/mL consistently correlated with complete depletion of asparagine from the plasma and CSF (102). A few years later, Berg et al. found that after administering a dose of 2,500 IU/m^2^ of IM PEG to rhesus monkeys, plasma asparagine remained undetectable at SAA levels of ≥0.1 IU/mL, though CSF asparagine levels were more variable (105). Other studies have challenged the 0.1 IU/mL benchmark. COG study AALL07P4 conducted extensive PK studies of patients treated with PEG and CAL-PEG and found that plasma and CSF asparagine began increasing to measurable levels in the 0.1–0.4 IU/mL range (28). In a study of 25 adults who were administered one dose of 2,000 IU/m^2^ PEG IV, pharmacodynamic modeling found that 0.2 IU/mL was the minimal SAA level that correlated with optimal serum asparagine depletion (106). Rizzari et al. evaluated patients treated on the AIEOP-BFM ALL 2009 study during induction. Enrolled patients received IV PEG 2,500 IU/m^2^ on days 12 and 26 of induction and serum and CSF samples obtained on days 33 and 45. They found that a majority of patients did not achieve complete asparagine depletion during the investigational period and CSF asparagine depletion correlated poorly with serum SAA levels (107). Additionally, a recent analysis using data from 482 children treated on the SJCRH Total XVI study combined with pharmacokinetic-pharmacodynamic modeling and simulation, Panetta et al. suggested higher SAA levels were needed. They found that the median SAA level needed to maintain CSF asparagine depletion (defined as below 1 μM) was 0.44 IU/mL (95% CI 0.2–0.99) (108). Conversely, Rizzari et al. reported that in 62 patients enrolled in the AIEOP ALL95 study given either one dose of ERW or native *E.coli* asparaginase either IV or IM, SAA levels <0.05 IU/mL were associated with undetectable plasma and CSF asparagine levels in most patients (109). Other studies using native *E.coli* asparaginase, recombinant *E. coli* asparaginase, and pegylated *E. coli* asparaginase have suggested ≥ 0.02 IU/mL is adequate to achieve asparagine depletion in the plasma and CSF (110, 111).

Despite the ongoing debate regarding the optimal nadir SAA levels, these assays have proven useful in the clinical setting. SAA levels can help distinguish an antibody mediated hypersensitivity reaction from a non-antibody mediated infusion reaction. While the symptoms of these reactions are largely overlapping, the ability to differentiate them is important to ensure optimal asparaginase therapy. In antibody mediated clinical hypersensitivity reactions, the anti-asparaginase antibodies characteristically bind to and cause the rapid elimination of asparaginase, rendering the drug ineffective (47). Patients who have had an antibody-mediated hypersensitivity reaction must therefore receive alternative, non-cross reactive asparaginase preparations (33). On the other hand, infusion reactions are due to non-antibody mediated mechanisms, including a sharp spike in ammonia after asparaginase (86, 112, 113) administration due to the rapid cleavage of asparagine into aspartic acid and ammonia (114). As infusion reactions occur in the absence of neutralizing antibodies patients do not need to change asparaginase preparations. After a clinical reaction, SAA levels can be used to determine if neutralizing antibodies are present (115) and if a change in asparaginase therapy is necessary.

SAA levels can also be used to identify rare patients who have neutralizing anti-asparaginase antibodies yet do not have symptoms of hypersensitivity, a scenario called silent inactivation or subclinical hypersensitivity. Similar to clinically overt hypersensitivity, asparaginase is rapidly destroyed in patients with silent inactivation, with a swift decline in SAA levels below therapeutic values after asparaginase administration. Patients with silent inactivation that are not identified and switched to an alternative asparaginase product have inferior outcomes, highlighting the importance of TDM (116). Additionally, in an effort to reduce clinical reactions and decrease the need to switch asparaginase preparations, especially in light of recent ERW drug shortages, premedication with antihistamines +/- corticosteroids prior to PEG administration has become increasingly common (57, 117). Although this strategy can mask the symptoms of an antibody mediated hypersensitivity reaction, most experts recommend TDM be done post-dosing in premedicated patients to ensure neutralizing antibodies are identified which enables the appropriate switching to an alternative asparaginase preparation. It should be noted, however, that the reported incidence of silent inactivation in patients treated with PEG varies, but most studies suggest rates of 0–8% (summarized in Marini et al.). Additionally, the risk of a masked antibody-mediated reaction post premedication also appears to be low, with only 1/68 patients in the Cooper et al. study and <1% in Marini et al. having evidence of neutralizing antibodies based on TDM *via* SAA levels (57, 117). Thus, the necessity for TDM with or without premeditation in patients without symptoms of a hypersensitivity reaction is a matter of ongoing debate.

In addition to identification of neutralizing antibodies, monitoring of SAA levels may offer an opportunity to individualize asparaginase dosing. A recent study by the Dutch Childhood Oncology Group (DCOG) ALL-11 investigated individualized dosing based on SAA trough levels. After three fixed doses of 1,500 IU/m^2^ during induction, patients in the medium-risk group (*N* = 243) were given 14 individualized doses of PEG with target trough levels of 0.1–0.25 IU/mL and patients in the standard-risk group (*N* = 108) received one individualized dose. After the 10th PEG dose, a median dose of 450 IU/m^2^ was able to sustain trough SAA levels in the goal range. Likewise, a majority of patients who switched to ERW due to clinical hypersensitivity or silent inactivation achieved goal trough SAA levels with reduced doses and/or less frequent administration (118). Individualized dosing based on SAA levels as demonstrated in this study could result in more efficient dosing of asparaginase. As most patients are able to achieve adequate SAA levels with reduced dosing, ultimately this could lead to less dose-dependent liver toxicity and, importantly, reduced healthcare utilization costs.

### Future of Asparaginase Therapy in ALL/LBL

Asparaginase is an established cornerstone of ALL/LBL therapy, however, opportunities for improvement remain. As clinical SAA assays are now widely available and can be used to guide asparaginase therapy, clarity around ideal and necessary SAA levels is still lacking. In part, this uncertainty is related to the fact that what is deemed the optimal therapeutic level is considered that which fully depletes plasma asparagine, yet measurement of plasma asparagine is fraught with technical issues which call into question their validity. Perhaps in future studies, SAA values could be correlated with outcome in large uniformly treated cohorts as a more direct assessment of SAA levels necessary for optimal outcomes.

Based on a number of studies, it is clear that omission of entire asparaginase courses is associated with inferior outcomes, particularly for higher risk patients. This is true for protocols that use either intermittent asparaginase dosing or continuous asparagine depletion. This highlights the necessity of having alternative asparaginase preparations for patients who develop antibody mediated hypersensitivity or silent inactivation to their first line asparaginase. Yet, ongoing manufacturing issues have led to frequent and prolonged shortages of ERW, which until recently was the only non-cross reactive asparaginase available to patients with hypersensitivity to PEG. The FDA approval of the recombinant Erwinia asparaginase (Rylaze) has improved this situation, providing an alternative, more readily manufactured product (38). However, ERW and Rylaze are both native, short-acting forms of asparaginase, requiring multiple doses over 2 weeks to achieve asparagine depletion comparable to PEG. Additionally, some data suggests that short acting EWR products are less effective at CSF asparagine depletion compared to PEG. Panetta et al. compared children given IM ERW or IV EWR to those given IV PEG. They observed no difference in median time of depletion following an IV dose of either 2,500 or 3,000 IU/m^2^ of PEG, but found the duration of CSF asparaginase depletion was significantly shorter longer than PEG after completing a course of ERW (administered every 72 h for 10 doses., either IV or IM, at a dose of either 30,000 or 42,000 IU/m^2^) (108). However, the study was limited by a low number of CSF samples in those receiving ERW. Alternative, long-acting versions of *Erwinia asparaginase* are therefore greatly needed. A pegylated *Erwinia asparaginase*, pegcrisantaspase, was developed and tested in a phase 2/3 trial ran by Jazz Pharmaceuticals and the COG. Unfortunately, three of the first four patients to receive pegcrisantaspase in this trial had either clinical hypersensitivity reaction with early drug clearance or silent inactivation. Immunogenicity studies revealed that the neutralizing antibodies were likely against the polyethylene glycol moiety, explaining the cross-reactivity with PEG (119). The occurrence of hypersensitivity to PEG due to antibodies against the polyethylene glycol moiety has been subsequently confirmed by a number of additional studies (43–45). Therefore, pegylation is likely not a feasible strategy for extending the half-life of *Erwinia asparaginase* used after hypersensitivity to PEG. Alternative strategies are being explored including a method termed PASylation in which the therapeutic protein is fused with a conformationally disordered 600 residue polypeptide rich in proline/alanine +/- serine (120). PASylation has been used to extend the half-life of a number of compounds (121) including immunostimulatory peptides (122), interferons (123), and urate oxidase (124). A PASylated *Erwinia asparaginase* is currently in the pre-clinical pipeline for Jazz pharmaceuticals^3^, offering hope for a long-acting *Erwinia asparaginase* in the future.

Another possible strategy to overcome issues with hypersensitivity that has shown promise is encapsulation of L-asparaginase in donor-derived erythrocytes (eryaspase). The encapsulated asparaginase remains active, cleaving asparagine that enters the encapsulating erythrocyte while ‘hiding’ the drug from the patient's immune system. The half-life of eryaspase is ~2 weeks based on pharmacokinetic studies in cancer populations including adults with pancreatic cancer (125, 126) and adults and children with ALL (127, 128). A recent phase 2 study (NOR-BRASPALL-2016, NCT01518517) enrolled children (*N* = 36) and adults (*N* = 2) with relapsed ALL and hypersensitivity to PEG and found that at the 14-day trough, 94.7% of patients had SAA levels >0.1 IU/mL and 71.1% were above 0.4 IU/mL. Overall, eryaspase was well-tolerated. Six of 36 patients had possible allergic reactions to eryaspase, three of whom had early clearance as determined by SAA levels (129). Ultimately, these early clinical studies led to FDA Fast Track designation of eryaspase in July of 2021.

## Conclusion

Asparaginase is a cornerstone of ALL/LBL therapy. While part of standard therapy for decades continual developments to improve pharmacokinetics, availability, and tolerability have contributed greatly toward improvement in patient outcomes. Approaches such as use of recombinant technology to overcome manufacturing issues, novel means of half-life extension and new modalities to reduce immunogenicity, have been significant recent advances in asparaginase therapy. Currently ongoing trials include a multi-institutional study aiming to define the impact of universal premedication on PEG associated hypersensitivity and a phase II trial seeking to determine the efficacy of levocarnitine and vitamin B complex in the treatment of PEG associated hyperbilirubinemia. The next horizons in asparaginase therapy include individualized dosing based on SAA levels, incorporation of pharmacogenomics to predict risk of toxicities and development of additional strategies to mitigate target toxicities.

## Author Contributions

LM and RR were responsible for the conception, content planning, and writing of the manuscript. Both authors contributed to the article and approved the submitted version.

## Conflict of Interest

LM served on advisory board and speakers bureau for Jazz Pharmaceuticals. RR served on advisory board for Jazz Pharmaceuticals and Servier Pharmaceuticals.

## Publisher's Note

All claims expressed in this article are solely those of the authors and do not necessarily represent those of their affiliated organizations, or those of the publisher, the editors and the reviewers. Any product that may be evaluated in this article, or claim that may be made by its manufacturer, is not guaranteed or endorsed by the publisher.

## Footnotes

^1^https://www.accessdata.fda.gov/scripts/drugshortages/dsp_ActiveIngredientDetails.cfm?AI=Asparaginase%20Erwinia%20Chrysanthemi%20(Erwinaze)&st=c&tab=tabs-1 Updated June 8, 2020. UFaDAFDS, 2022 AF.

^2^RYLAZE™ (asparaginase erwinia chrysanthemi (recombinant)-rywn) [package insert]. Leinster IJPILJ.

^3^ https://www.jazzpharma.com/science/pipeline/
